# A Case of Inoperable Malignant Insulinoma with Resistant Hypoglycemia Who Experienced the Most Significant Clinical Improvement with Everolimus

**DOI:** 10.1155/2013/636175

**Published:** 2013-05-08

**Authors:** Emre Bozkirli, Okan Bakiner, Huseyin Abali, Cagatay Andic, Ali Fuat Yapar, Fazilet Kayaselcuk, Eda Ertorer

**Affiliations:** ^1^Division of Endocrinology and Metabolism, Adana Medical Center, Baskent University School of Medicine, Dadaloglu Mah. Serin Evler 39, Sok. No. 6 Yuregir, 01250 Adana, Turkey; ^2^Division of Medical Oncology, Adana Medical Center, Baskent University School of Medicine, Dadaloglu Mah. Serin Evler 39, Sok. No. 6 Yuregir, 01250 Adana, Turkey; ^3^Division of Radiology, Adana Medical Center, Baskent University School of Medicine, Dadaloglu Mah. Serin Evler 39, Sok. No. 6 Yuregir, 01250 Adana, Turkey; ^4^Division of Nuclear Medicine, Adana Medical Center, Baskent University School of Medicine, Dadaloglu Mah. Serin Evler 39, Sok. No. 6 Yuregir, 01250 Adana, Turkey; ^5^Division of Pathology, Adana Medical Center, Baskent University School of Medicine, Dadaloglu Mah. Serin Evler 39, Sok. No. 6 Yuregir, 01250 Adana, Turkey

## Abstract

Metastatic insulinomas may sometimes present with recurrent life-threatening hypoglycemia episodes. Such patients usually fail to respond to various therapeutic agents which causes constant dextrose infusion requirement. Herein, we present a resistant case of inoperable malignant insulinoma who was treated with many therapeutic agents and interventions including somatostatin analogues, Yttrium-90 radioembolization, everolimus, radiotherapy, and chemoembolization. Close blood sugar monitorization during these therapies showed the most favourable response with everolimus. Everolimus treatment resulted in rapid improvement of hypoglycemia episodes, letting us discontinue dextrose infusion and discharge the patient. However, experience with everolimus in such patients is still limited, and more precise data can be obtained with the increasing use of this agent for neuroendocrine tumours.

## 1. Introduction

Insulinomas are the most common functioning pancreatic neuroendocrine tumours (PNETs) with an annual incidence of one to five cases per million. Although they are usually benign solitary tumours, approximately 10% of the cases have distant metastases at diagnosis [1–4]. According to the World Health Organization (WHO), the only criterion for malignancy is the presence of metastases and malignant insulinomas are associated with a higher risk of recurrence and mortality [5]. Autonomous production of excessive amounts of insulin resulting in life-threatening hypoglycemia is the classical feature of the disease. A 72-hour supervised fasting study demonstrating hyperinsulinemia and simultaneous nonsuppressed C-peptide levels during biochemically proven hypoglycemia is the recommended diagnostic procedure; however, most of the patients experience hypoglycemia within the first 24 hours [6]. 

Surgery is the first choice of treatment for benign and malignant insulinomas [7]. However, some malignant insulinomas may be unresectable and treatment options are limited for these patients. Diazoxide, beta-blockers, diphenylhydantoin, and somatostatin analogues may be helpful medical treatment options in terms of hypoglycemia control [8–10]. Systemic chemotherapy, radioembolization, chemoembolization, radiotherapy, and peptide receptor radionuclide therapy can be tried as antitumour therapies [9, 11–13]. Everolimus is one of the mammalian target of rapamycin (mTOR) inhibitors which is increasingly used as a new class of agents for the treatment of PNETs [14, 15]. It is thought to have effects on both tumour growth and glycemic regulation for insulinomas [16–18].

## 2. Case

A 61-year-old woman with history of recurrent life-threatening hypoglycemic episodes was referred to our hospital with the suspicion of an insulinoma. She was living in a rural area and her medical history did not reveal any systemic disease and drug use. Hypoglycemic episodes were reported to begin a year ago before admission and were becoming more frequent and severe by time. Diagnostic work-up for the exclusion of other potential causes of hypoglycemia was completed at the center which she was referred from. 

On admission, she was reported to be on continuous intravenous (IV) dextrose infusion for the last two weeks for preventing the life-threatening episodes of hypoglycemia. Following hospitalisation at our clinic, dextrose infusion was stopped and fifteen minutes after she presented with neurological symptoms of hypoglycemia. Her simultaneous plasma glucose was 41 mg/dL with inappropriately high plasma insulin and C-peptide levels; 82.2 *μ*IU/mL (2.6–25 *μ*IU/mL) and 3.02 pmol/L (0.15–0.30 pmol/L), respectively. The diagnosis was confirmed as endogenous autonomous hyperinsulinism and further investigation to search for an insulinoma was begun. 

**Figure 1:**
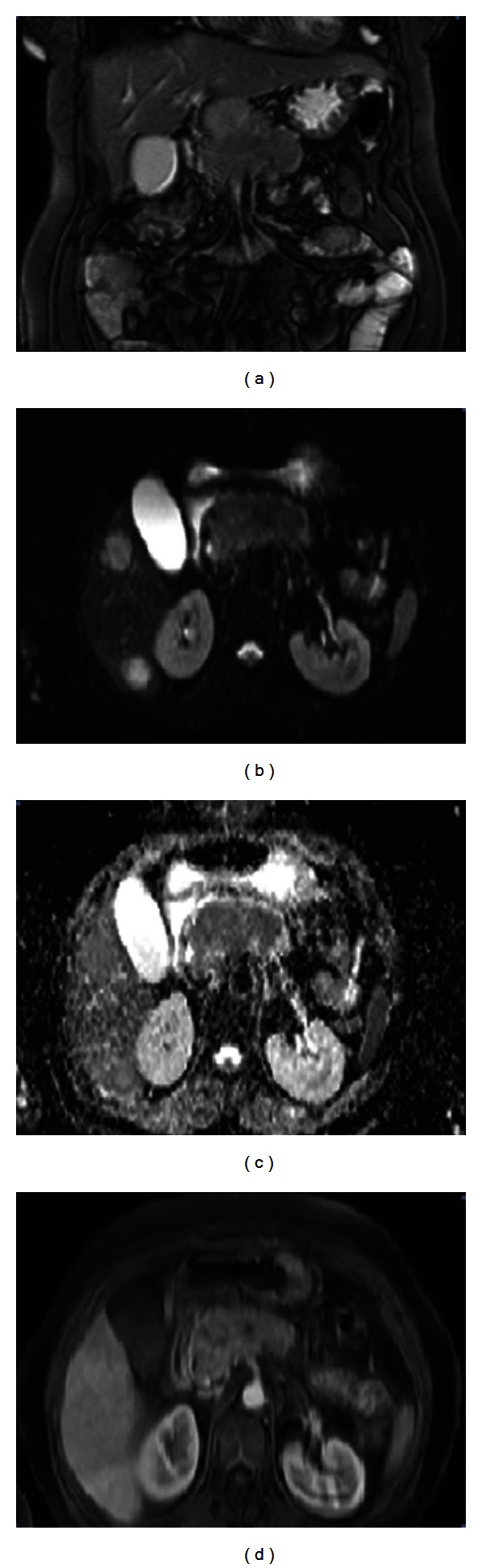
Coronal and axial images from initial abdomen MRI showing the primary tumour found between head and corpus of pancreas and invasion of the adjacent large vessels.

**Figure 2:**
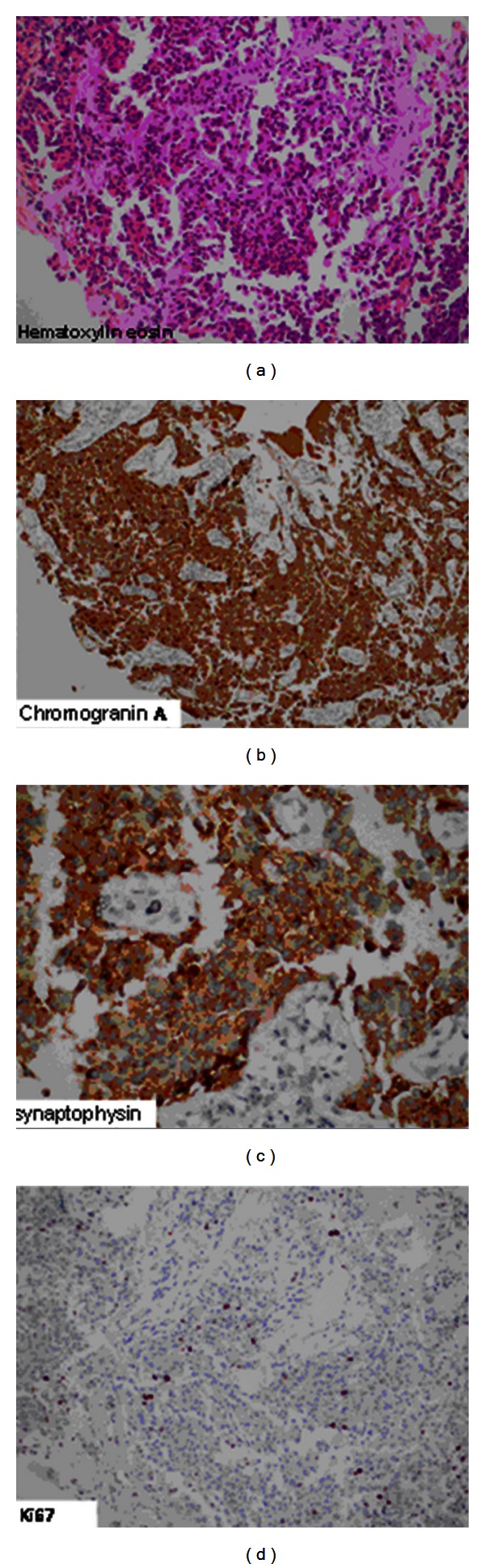
Pathologic specimens of liver metastases, Hematoxylin eosin, chromogranin A and Ki 67 with ×100 magnification, Synaptophysin with ×200 magnification.

Magnetic resonance imaging (MRI) of her abdomen demonstrated a 74 × 33 mm primary tumour causing enlargement in the body of pancreas with multiple lymph nodes near portal hilus around celiac trunk and multiple metastatic lesions in both lobes of the liver with the largest one 5 cm in diameter (Figure 1). Histological examination of the liver lesions was reported as neuroendocrine tumour metastasis with positive immunohistochemical staining for chromogranin and synaptophysin and a Ki-67 index below 2% (Figure 2).

Indium-111 pentetreotide scan (OctreoScan) demonstrated intense uptake of the radiotracer in primary pancreatic tumour, in multifocal liver lesions and regional lymph nodes. She was considered as inoperable because of the invasion of the large vessels adjacent to the primary tumour and widespread distribution of liver metastases (Figure 1). The patient was discussed at our multidisciplinary tumour board and she was considered inoperable and medical therapy was advised.

**Figure 3:**
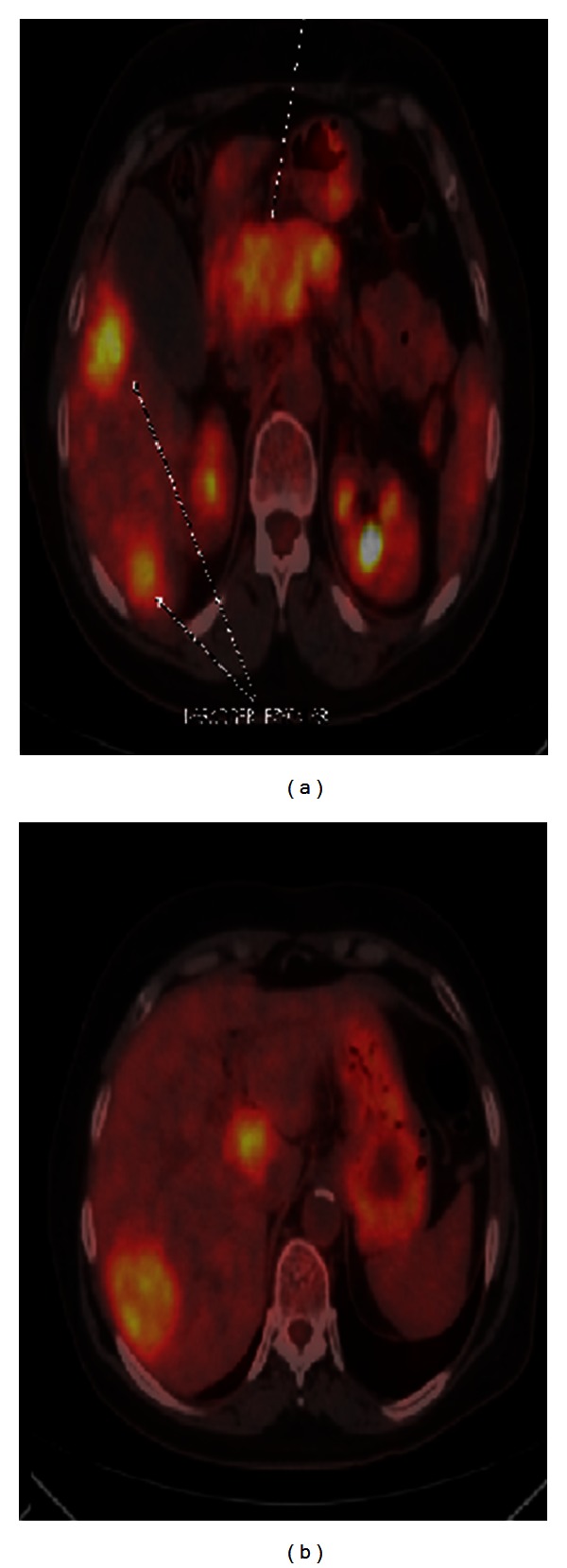
Intense involvement of Yttrium-90 in pancreas and hepatic metastases.

Subcutaneous Short acting somatostatin analogue, octreotide, was administered, but no clinical improvement was observed in spite of dose increment up to 200 *μ*g three times daily. Radioembolization of the liver metastatic lesions was performed concomitantly by injecting 50 mCi (1.85 GBq) Yttrium-90 labeled resin microspheres (Sir-spheres) via hepatic artery (Figure 3). 

After a month of in-patient treatment since radioembolization with on-going subcutaneous Short acting octreotide therapy, the patient still required continuous and constant intravenous dextrose infusion and could not be discharged. Although her insulin and C-peptide levels were lower during hypoglycemia, they were still above the reference limits (61.9 *μ*IU/mL and 1.97 pmol/L, resp.). 

The miserable clinical state of this malignant inoperable insulinoma patient led us to search for the limited medical literature on this topic again. A decision was made in favour of withdrawing octreotide and giving her oral everolimus treatment with radiotherapy to the primary tumour, which was considered as a significant source of endogenous insulin secretion.

 Oral everolimus treatment at a dose of 10 mg once daily and concomitant 15 fractioned doses and 45-Gray radiotherapy were given. The patient exhibited immediate favourable response to the new treatment that was clearly documented with blood glucose monitoring. Her continuous requirement for dextrose infusion began to decrease on the fifth day of everolimus and dextrose infusion was completely withdrawn on the seventh day of everolimus. She became relatively well in condition and could manage to stay without dextrose infusion for hours. However, discharge was again not possible due to the life-threatening hypoglycemic episodes that happened unexpectedly. During one of these episodes, her blood glucose was found to be 32 mg/dL with relatively high simultaneous insulin and C-peptide levels 13.4 *μ*IU/mL and 0.86 pmol/L, respectively. 

**Figure 4:**
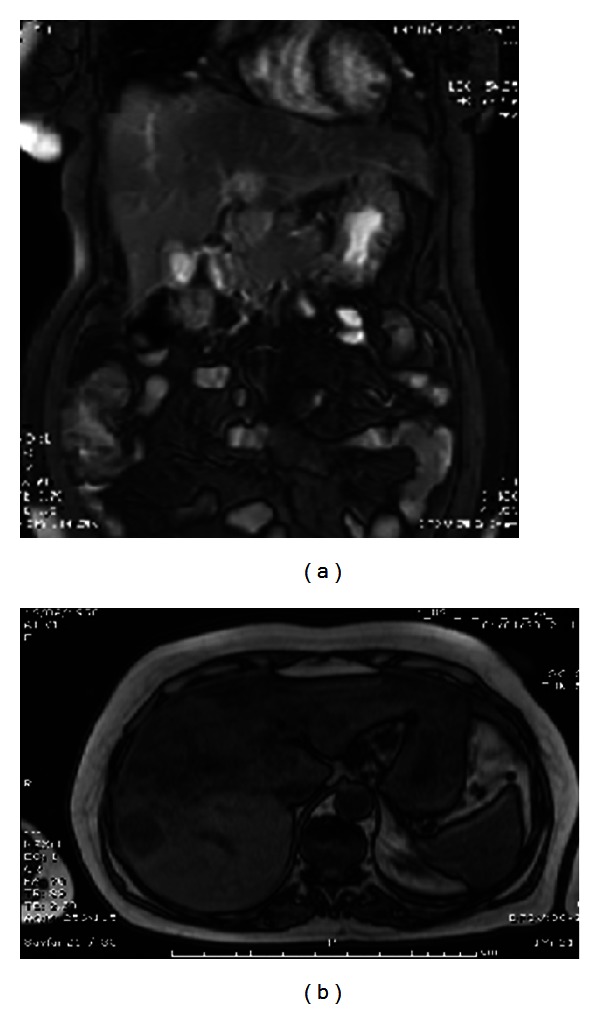
Coronal and axial images from control MRI.

At the end of her second month of hospitalisation, while she was doing pretty well on everolimus 10 mg/day, an MRI of abdomen was reperformed. It revealed regression in primary tumour and in the lesions located at the left lobe of liver, but two metastatic masses at the right liver lobe were reported to remain unchanged (Figure 4).

Depending on the fact that she still had a high tumour burden and although rarely and still experienced life-threatening unexpected hypoglycemic episodes against all the interventions mentioned above and continuing everolimus treatment, we decided for alternative modalities of therapy. Thus, chemoembolization with 5 fluorouracil and doxorubicin + DC beat (300–500) microparticles was performed after selective catheterization of right lobe of the liver. Excluding the hypoglycemic episode that happened on the day of chemoembolization, she did not experience any hypoglycemia thereafter. On her last hypoglycemic episode, her plasma glucose, insulin, and c-peptide levels were, 37 mg/dL, 17.5 *μ*IU/mL, and 1.19 pmol/L, respectively. She had been followed only on everolimus for a week and was discharged with it. 

Maybe because of being an illiterate woman from a distant rural part of our country, she did not attend at control visits during the following four months. On our telephone calls, her relatives reported that she was fine and experienced no hypoglycemic episode as long as she took her everolimus regularly.

## 3. Discussion

Herein, we reported a very rare case of malignant insulinoma whose treatment was really a challenge. The widespread tumour disabled performance of surgical treatment which was the first treatment of choice. Short acting subcutaneous octreotide, Y-90 microsphere radioembolization to liver metastases, radiotherapy to primary tumour, and chemoembolization to hepatic metastases were all inconclusive. The patient demonstrated immediate and clear response only to oral everolimus in terms of hypoglycemic episode management.

 Surgery is the first choice of therapy for resectable malignant insulinomas, while medical therapy is indicated for patients with unresectable tumours to control insulin hypersecretion and hypoglycemia. Diazoxide; an agent which suppresses the release of insulin from insulinoma cells via opening ATP-sensitive potassium channels, helps to prevent hypoglycaemia [8]. Short acting somatostatin analogue; octreotide is another medical option to suppress excess insulin secretion. Both of these agents can be used both during the preoperative preparation period of benign and malign insulinomas, and for preventing hypoglycaemia of insulinomas with unidentified location. Diazoxide is unavailable on the market in our country, so we started our treatment with Short acting octreotide. However, response to this somatostatin analogue may differ according to the presence of various subtypes of somatostatin receptor (sst) on insulinoma cells. Octreotide binds predominately to somatostatin receptor subtype 2 (sst_2_). The absence of these receptors on insulinoma cells of an individual may result in aggravation of hypoglycemia when he is treated with octreotide. This effect may be attributed to the inhibition of insulin-antagonistic hormones such as growth hormone and glucagon by somatostatin [19]. Vezzosi et al., in their insulinoma series, reported a 50% success rate with octreotide in terms of hypoglycaemia. However, their patients were all benign insulinomas with positive immunostaining for sst_2_ [20]. In our case, although the malignant intra-abdominal lesions demonstrated intense uptake during OctreoScan, the hormonal response to octreotide treatment was poor. This finding made us think that her tumour might probably express somatostatin receptor subtypes other than subtype 2.

 In our experience, octreotide failed to control hypoglycemia episodes, therefore, radioembolization for hepatic metastases was planned as a second line therapy. Limited studies investigating the use of Y-90 radioembolization for metastatic neuroendocrine tumours reported an overall response rate between 32 and 90% [21]. Our patient's constant dextrose requirement continued after radioembolization although her insulin and C-peptide levels decreased. The precise effects of radioembolization are predicted to occur in three months but because of the severity of the patient's clinic she was discussed again at the tumour board. Based on the promising results with everolimus she was planned to take everolimus and a rapid response was observed in blood sugar monitorization with the initiation of everolimus treatment. Having done a radioembolization to liver metastases, we thought that the primary tumour was still a significant source of endogenous insulin and decided to irradiate it externally since we wanted to be sure that she would be free of hypoglycemia episodes after discharge. We felt insecure only with everolimus in out-patient setting instead of conventional chemotherapy since she was coming from a distant and undeveloped part of our country. Furthermore studies demonstrating the efficacy of everolimus as a radiosensitizer contributed to this decision [22]. We observed no unusual side effect with concomitant use of external radiotherapy to primary in pancreas and everolimus. Rapid response in the mean of hypoglycemia control was observed after therapy. This finding was attributed to everolimus because effects of radiotherapy are expected to occur in long time. Everolimus probably decreases insulin production and release from the pancreatic beta cells through the AMP-activated protein kinase (AMPK)/c-Jun N-terminal kinase (JNK)/FoxO pathway and it probably induces peripheral insulin resistance [9, 16]. In a series consisting of four patients, Kulke and coworkers reported success after everolimus treatment in the mean of discontinuation of administering glucose and diazoxide [16]. However in our case during the follow-up period chemoembolization to hepatic metastases had to be performed for reducing tumour burden because of on-going less frequent hypoglycemia episodes. 

As a conclusion many therapeutic approaches like octreotide therapy, radioembolization, radiotherapy, and chemoembolization were performed for our resistant malign insulinoma patient and the most favourable response in terms of symptom control was obtained with everolimus shown with close blood glucose monitoring. Furthermore, we did not observe any side effect while continuing everolimus during radiotherapy. Fortunately insulinoma patients in such severity are very rare and mTOR inhibitors like everolimus may be promising, but studies with more patients are required to support this proposal.

## Abbreviations

PNETs: Pancreatic neuroendocrine tumours
mTOR: Mammalian target of rapamycin
IV: Intravenous
MRI: Magnetic resonance imaging
sst: Subtypes of somatostatin receptor.
